# Self-harm and violent criminality among young people who experienced trauma-related hospital admission during childhood: a Danish national cohort study

**DOI:** 10.1016/S2468-2667(17)30094-4

**Published:** 2017-06-01

**Authors:** Roger T Webb, Sussie Antonsen, Matthew J Carr, Louis Appleby, Carsten B Pedersen, Pearl L H Mok

**Affiliations:** a Centre for Mental Health & Safety, Division of Psychology & Mental Health, School of Health Sciences, Faculty of Biology, Medicine and Health, The University of Manchester, Manchester, UK; b Manchester Academic Health Science Centre (MAHSC), Manchester, UK; c National Centre for Register-based Research, Department of Economics and Business Economics, Aarhus University, Aarhus, Denmark; d Centre for Integrated Register-based Research, CIRRAU, Aarhus University, Aarhus, Denmark

## Abstract

**Background:**

Development of a better understanding of subsequent pathways for individuals who experienced trauma during childhood might usefully inform clinicians and public health professionals regarding the causes of self-harm and interpersonal violence. We aimed to examine these risks during late adolescence and early adulthood among people admitted to hospital following injuries or poisonings during their childhood.

**Methods:**

This national cohort study included Danish people born between Jan 1, 1977, and Dec 31, 1997, and was linked to the National Patient Register and Psychiatric Central Research Register to identify all people exposed to hospital admissions for injuries or poisonings due to self-harm, interpersonal violence, or accidents before their 15th birthday. Linkage to these two registers and to the National Crime Register enabled ascertainment of self-harm and violent offending, respectively, as adverse outcomes at ages 15–35 years. Sex-specific incidence rate ratios (IRRs; relative risks) and cumulative incidence percentage values (absolute risks) were estimated. The confounding influence of parental socioeconomic status was also explored.

**Findings:**

1 087 672 Danish people were included in this study. The prevalence of any trauma-related hospital admission was 10% (105 753 per 1 087 672; males: 64 454 [11%]; females: 44 299 [8%]) and for both sexes, accident was by far the most prevalent of the categories assessed (males: 59 011 [11%]; females: 40 756 [8%]). Similar patterns of increased risk for self-harm and violent criminality were observed in both sexes, although the IRRs were consistently and significantly larger in women (self-harm: IRR 1·94 [95% CI 1·85–2·02]; violent criminality: 2·16 [1·97–2·36]) than in men (self-harm: 1·61 [1·53–1·69]; violent criminality: 1·58 [1·53–1·63]). Confounding by parental socioeconomic status explained little of the increased risks observed. For young adult men, the highest absolute risk observed was for violent offending among individuals admitted to hospital for interpersonal violence injury during childhood (cumulative incidence 25·0% [95% CI 21·2–28·9]). For young adult women, absolute risk was highest for repeat self-harm among those admitted to hospital following self-harm during childhood (cumulative incidence 21·4% [95% CI 19·8–23·1]). More frequent trauma-related hospital admissions in childhood, and being admitted multiple times for more than one reason, conferred substantial risk increases among young people, with especially steep gradients of this nature observed among women.

**Interpretation:**

Trauma-related hospital admission early in life could be a useful marker for childhood distress that subsequently predicts internalised and externalised destructive behaviours among youths and young adults and might provide a timely opportunity for initiating family-oriented interventions.

**Funding:**

European Research Council.

## Introduction

Trauma-related hospital admission following serious injuries and poisonings, whether self-inflicted, perpetrated by other people, or occurring accidentally, can be highly distressing experiences for children and their families. Furthermore, these episodes predict short-term and long-term increases in risk of suicide and other causes of premature death,^1^, ^2^, ^3^, ^4^ and they also absorb scarce health-care resources disproportionately.^4^ To inform the coordinated development of multiagency initiatives to reduce adverse outcome risks among psychosocially vulnerable young people, it is therefore necessary to better understand the long-term trajectories of children who require hospital admission following major injuries or poisonings, to help to ensure their safe progression through to healthy adult maturity. These trauma-related hospital admissions have been described as salient so-called teachable moments, providing an opportunity to deliver considered interventions that could potentially prevent future harmful behaviours.^5^

A paper published in 2015 reported on a large longitudinal investigation of hospital admissions for violent, drug-related or alcohol-related, self-inflicted, and accidental injury among adolescents aged 10–19 years in England.^3^ The study indicated strong links between this exposure and risks of death and emergency readmission within 10 years of the index hospitalisation episode. Two previous investigations reporting results from large routinely collected datasets in New Zealand have examined subsequent risks of assaultive injury,^6^ self-injury, and suicide,^7^ without specifying an age range for experiencing trauma-related hospital admission. We examined such hospital admissions that occurred specifically during childhood, to establish the longer-term trajectories of this particularly vulnerable group of young people.

**Research in context**

**Evidence before this study**

We searched for article titles published in English in MEDLINE and Embase up until Feb 24, 2017, that included the following combination of terms: “child” or “youth” or “adolesc” AND “hospitali” or “admitted” or “admission” AND “trauma” or “self-harm” or “self-inflict” or “self-injur” or “self-poison” or “suicid” or “violen” or “assault” or “accident” or “death” or “die” or “mortality”. We discovered that the existing evidence-base for this topic was limited. Previous national registry studies have examined links between history of trauma-related hospital admission and future risks of self-harm and suicide, assaultive injury, emergency readmission, and premature death. These studies have consistently shown strong associations, but several important research questions remain unanswered, including the long-term trajectories of people who experienced trauma-related hospital admissions during childhood, assessment of self-harm versus violent offending risks in the same study cohort, and cumulative risk estimation.

**Added value of this study**

We did a national cohort study of more than 1 million people. We identified all trauma-related hospital admissions during childhood through to the 15th birthday and examined adverse outcomes between mid-adolescence and age 35 years. We compared risks for internalised versus externalised violence, and we generated measures of sex-specific absolute risks in this population by deriving estimates of cumulative incidence values that accounted for competing risks. Around one in seven men admitted to hospital during childhood following self-harm and one in four due to interpersonal violence will be convicted for committing a violent crime by age 35 years. About one in five women admitted to hospital after an episode of self-harm or interpersonal violence during childhood will be admitted to hospital again following self-harm between their 15th and 35th birthdays.

**Implications of all the available evidence**

The psychosocial wellbeing of individuals who experienced trauma-related hospital admission while growing up warrants careful consideration in the development of comprehensive strategies to address internalised and externalised violence in young people. National clinical guidelines for provision of psychosocial assessment target single problems such as self-harm, but they could be usefully broadened to encompass other adverse events such as hospital admission of children following episodes of interpersonal violence or serious accidents. Trauma-related hospital admission of a child might present an important opportunity to implement a family-oriented intervention in the hospital setting, with the proactive purpose of reducing future risk of harmful or self-destructive behaviours. Particularly, close monitoring and robust support are indicated for young women who were admitted to hospital as children on more than one occasion following trauma and for those who were admitted post trauma for multiple reasons during their childhood.

Published reports have tended to focus on a single adverse outcome, such as youth suicide,^8^ whereas we set out to harness the potential of national Danish registers to examine the longer-term trajectories of affected children. We investigated self-harm and violent criminality as adverse outcomes because self-directed and externalised violence are associated harmful behaviours that share common causal factors. A Swedish national registry study from 2006 reported a five times increasd risk of violent crime conviction among people with a history of hospital-treated self-harm, with an independent doubled risk after adjustment for psychiatric comorbidity and environmental factors.^9^ The combined societal costs of these two related deleterious behaviours are immense,^10^ prompting calls for concerted action to tackle them in unison.^11^

The aims of this national cohort study were: (1) to estimate the relative and absolute risks of self-harm and violent criminality among youths and young adults who experienced hospital admission due to injuries and poisonings during their childhood; (2) to compare these estimates by sex and by cause of hospital admission—self-harm, interpersonal violence, or accident; (3) to assess confounding by parental socioeconomic status (SES); and (4) to evaluate effect modification by frequency of and multiple reasons for trauma-related hospital admission during childhood. We anticipated observing especially increased risks among individuals admitted to hospital as children following interpersonal violence or self-harm versus the reference category of people who had no experience of trauma-related hospital admissions during childhood. We also examined exposure to hospital admission following accidental injury or poisoning as an additional comparison. We acknowledge that a sizeable proportion of admissions following accidents might not have been anywhere near as traumatising to the affected children as those that followed internalised or externalised violence.

## Methods

### Study design and participants

Since 1968 the Civil Registration System has registered all Danish residents^12^ by capturing date and place of birth, sex, and continuously updated vital status information. Its mandatory unique personal identification numbering system enables complete and accurate linkage to health-related administrative registers as well as similarly comprehensive parent-offspring linkage. The study cohort consisted of all people born in Denmark from Jan 1, 1977, to Dec 31, 1997, who still resided in Denmark at their 15th birthday, and whose parents were both Danish-born, thereby accounting for increased risks of self-harm and violent criminality linked with first and second generation immigrant status.^13^ Furthermore, the study registers provided less complete information about experience of trauma-related hospital admissions during childhood for first-generation immigrants, because only those episodes experienced during residence in Denmark are captured in the nationwide administrative registers.

In this register-based study, consent to participate from cohort members was not needed. Cohort members were followed up between their 15th and 35th birthdays. Follow-up ended at the first occurrence of the adverse outcome of interest, emigration, death, or the final observation date of Dec 31, 2012, whichever date was earliest. Self-harm and violent criminal offending risks were assessed from the 15th birthday and onwards in the 1977–97 birth cohort. Therefore, the earliest outcome ascertainment date was Jan 1, 1992, and the latest was Dec 31, 2012, meaning that only those cohort members born during 1977 could provide complete follow-up information through to their 35th birthdays.

Approval to conduct the study was given by the Danish Data Protection Agency, and data access was granted by the State Serum Institute and by Statistics Denmark.

## Procedures

### Classification of trauma-related hospital admissions during childhood

Hospital admissions for injuries or poisonings between cohort members' births and their 15th birthdays were identified on the basis of “reason for contact” coding recorded in the National Patient Register^14^ and according to the 8th^15^ and 10th^16^ revisions of the International Classification of Diseases (ICD) as recorded in the National Patient Register and Psychiatric Central Research Register,^17^ as follows: (1) self-harm (“reason for contact”=4; complex ICD-based algorithm published previously);^18^ (2) interpersonal violence (“reason for contact”=3; ICD-8: E960-E969; ICD-10: X85-Y09); and (3) accident (“reason for contact”=2; ICD-8: E800-E949; ICD-10: V01-X59). In delineating individuals who were admitted to hospital following self-harm during childhood, we only included episodes in which the child had reached their tenth birthday on the admission date. Emergency room and ambulatory care visits could not be included in any of the exposure classifications because they were only captured in the two hospital registers from 1995. Thus, the stringent exposure classification included only infants and children who were admitted to hospital, thereby providing a proxy for more serious episodes that would usually constitute a more traumatic experience for affected children.

### Ascertainment of adverse outcomes between the 15th and 35th birthdays

To ascertain admissions following self-harm occurring beyond the 15th birthday, we used the aforementioned previously reported complex algorithm^18^ that entailed linkage to and extraction of data from the Psychiatric Central Research Register and the National Patient Register. The study cohort was also linked to the National Crime Register^19^ to identify all violent crime convictions, including homicide, assault, robbery, aggravated burglary or arson, possessing a weapon in a public place, violent threats, extortion, human trafficking, abduction, kidnapping, rioting and other public order offenses, terrorism, and sexual offences.

### Adjustment for parental SES

This potentially important confounding influence was measured for the entire study population at cohort members' 15th birthdays according to income quintile, highest educational attainment level (primary school, high school or vocational training, or higher education) and employment status (employed, unemployed, or outside the workforce for other reasons). As a sensitivity analysis, we compared models adjusted for parental SES measured at ages 5 years, 10 years, and 15 years versus those adjusted for parental SES measured at age 15 years only. These data were extracted from the Integrated Database for Labour Market Research.^20^

### Statistical analysis

Data were analysed with SAS statistical software version 9.4 (SAS Institute Inc, Cary, NC, USA). Sex- specific analyses are reported throughout this report due to substantial differences that we have previously shown between male and female incidence rates for self-harm and violent criminal offending among Danish youths and young adults.^21^ Incidence rate ratios (IRRs) were estimated by fitting log-linear Poisson regression models^22^ adjusted for age group and calendar year as time-dependent variables. Parental SES was adjusted for using time-fixed variables. The outcomes, self-harm and violent crime conviction, were examined separately according to time to first event since the 15th birthday. Thus, the estimated IRRs pertain to these first events only as repeat events were not assessed. The reference category for all IRRs reported was people who did not experience a hospital admission for injury or poisoning between their birth and 15th birthday. p values and 95% CIs were calculated from likelihood ratio tests. From competing risks survival analysis,^23^ cumulative incidence (absolute risk) was calculated as the probability of experiencing the specific outcome of interest, taking into account emigration or death. As with the IRR estimates, these cumulative incidence values were calculated separately for each childhood hospital admission exposure type: self-harm, interpersonal violence, or accident.

### Role of the funding source

The funder of the study had no role in the study design, data collection, data analysis, and data interpretation, or writing of the report. The corresponding author had full access to the data and had final responsibility to submit for publication.

## Results

1 087 672 Danish people were enrolled in this study. Table 1 shows the sex-specific prevalence values for trauma-related hospital admissions between cohort members' births and their 15th birthdays, with estimates reported separately by reason for hospital admission. The prevalence of any trauma-related hospital admission was 10% (105 753 per 1 087 672; men: 64 454 [11%]; women: 44 299 [8%]) and for both sexes, accident was by far the most prevalent of the categories assessed (men: 59 011 [11%]; women: 40 756 [8%]. All estimated prevalence values were substantially greater for men than for women, as indicated by the male to female prevalence rate ratios (PRRs), except for hospital admission following self-harm (PRR 0·58 [95% CI 0·55–0·61]). The rarest reason for hospital admission was interpersonal violence, and this category also had the largest observed male:female PRR (1·79 [95% CI 1·59–2·02]).

**Table 1:** Prevalence and male to female prevalence rate ratios by reason for trauma-related hospital admission before the 15th birthday

	**Prevalence in men (n=557 976)**	**Prevalence in women (n=529 696)**	**Male: female prevalence**
Self-harm	2371 (<1%)	3896 (1%)	0·58 (0·55–0·61)
Interpersonal violence	781 (<1%)	414 (<1%)	1·79 (1·59–2·02)
Accident	59 011 (11 %)	40 756 (8%)	1·37 (1·36–1·39)
Any trauma-related hospital admission	61 454 (11%)	44 299 (8%)	1·32 (1·30–1·33)

Data are n (%) or rate ratio (95% CI).

Table 2 shows the IRRs for adverse outcomes at ages 15–35 years linked with trauma-related hospital admission at least once during childhood. In men and women, there was a significant increase in risk for both adverse outcomes. Essentially the same patterns of increased risk were observed in both sexes, although the IRRs were consistently and significantly larger in women (self-harm: IRR 1·94 [95% CI 1·85–2·02]; violent criminality: 2·16 [1·97–2·36]) than in men (self-harm: 1·61 [1·53–1·69]; violent criminality: 1·58 [1·53–1·63]). The observed associations were only slightly attenuated following adjustment for parental SES measured at cohort members' 15th birthdays. Additional adjustment for parental SES measured at ages 5 years, 10 years, and 15 years, compared with adjustment for parental SES measured at age 15 years only, made almost no difference to the degree of attenuation observed (data not shown).

**Table 2:** Incidence rate ratios (IRRs) for self-harm and violent criminality at ages 15–35 years, linked with any trauma-related hospital admission before 15th birthday

		**People with adverse outcome (n)**	**Incidence rate per 10 000 person-years**	IRR 1*(95% CI)	**IRR 2**†**(95% CI)**
**Men**					
Self-harm					
	Any trauma-related hospital admission	1806	30·9	1·61 (1·53–1·69)	1·50 (1·42–1·58)
	No trauma-related hospital admission (ref)	8871	18·5	1·00	1·00
Violent crime conviction					
	Any trauma-related hospital admission	4306	76·0	1·58 (1·53–1·63)	1·49 (1·44–1·54)
	No trauma-related hospital admission (ref)	21 662	46·0	1·00	1·00
**Women**					
Self-harm					
	Any trauma-related hospital admission	2343	58·8	1·94 (1·85–2·02)	1·85 (1·77–1·94)
	No trauma-related hospital admission (ref)	13 028	28·4	1·00	1·00
Violent crime conviction					
	Any trauma-related hospital admission	579	14·1	2·16 (1·97–2·36)	1·91 (1·73–2·10)
	No trauma-related hospital admission (ref)	2815	6·0	1·00	1·00

* Adjusted for age band and calendar year period.

† Adjusted for age band and calendar year period, and also for parental socioeconomic status measured at the 15th birthday.

The IRRs presented in table 3 are reported according to reason for hospital admission during childhood (self-harm, interpersonal violence, or accident) in relation to the two adverse outcomes at ages 15–35 years: self-harm and violent criminality. Hospital admission during childhood following episodes of self-harm or interpersonal violence was strongly associated with later risks of self-harm and violent offending (table 3). Much weaker links were seen between childhood hospital admission following an accident and the two adverse outcomes through older adolescence and young adulthood (table 3). Again, the patterns of increased risk as indicated by the IRRs were similar for both sexes, although stronger associations were noted in women than in men. The increase in violent offending risk was almost ten-fold among women who experienced hospital admission following interpersonal violence during childhood (IRR 9·85 [95% CI 6·67–13·92]).

**Table 3:** Incidence rate ratios (IRRs) and cumulative incidence values (%) for self-harm and violent offending at ages 15–35 by reason for trauma-related hospital admission before the 15th birthday

		**People with adverse outcome (n)**	**Incidence rate per 10 000 person-years**	IRR*(95% CI)	**Cumulative incidence**†**(95% CI)**
**Men**					
Self-harm‡					
	Self-harm§	110	50·8	2·24 (1·84–2·69)	7·5% (5·7–9·7)
	Interpersonal violence	54	65·6	3·14 (2·37–4·05)	9·4% (7·1–12·0)
	Accident	1688	30·1	1·54 (1·46–1·62)	5·0% (4·6–5·3)
	No trauma-related hospital admission (ref)	8871	18·5	1·00	3·2% (3·1–3·2)
Violent crime conviction					
	Self-harm	230	110·3	1·92 (1·69–2·19)	13·7% (11·7–15·8)
	Interpersonal violence	149	196·5	3·82 (3·24–4·47)	25·0% (21·2–28·9)
	Accident	4012	73·8	1·50 (1·45–1·56)	10·4% (10·0–10·8)
	No trauma-related hospital admission (ref)	21 662	46·0	1·00	6·8% (6·7–6·9)
**Women**					
Self-harm‡					
	Self-harm§	657	248·0	6·60 (6·10–7·14)	21·4% (19·8–23·1)
	Interpersonal violence	54	152·2	4·16 (3·14–5·37)	18·3% (13·5–23·6)
	Accident	1789	47·9	1·47 (1·40–1·55)	6·5% (6·1–7·0)
	No trauma-related hospitalisation (ref)	13 028	28·4	1·00	4·0% (4·0–4·1)
Violent crime conviction					
	Self-harm	163	55·4	6·41 (5·45–7·48)	6·4% (5·2–7·8)
	Interpersonal violence	29	77·8	9·85 (6·67–13·92)	8·4% (5·7–11·6)
	Accident	430	11·2	1·58 (1·42–1·74)	1·5% (1·3–1·6)
	No trauma-related hospital admission (ref)	2815	6·0	1·00	0·9% (0·9–0·9)

* Adjusted for age band and calendar year period.

† Measures the probability or risk of experiencing the outcome of interest by the 35th birthday.

‡ Self harm as an outcome (15–35 years).

§ Self harm as a reason for hospital admission (before age 15 years).

**Figure 1:**
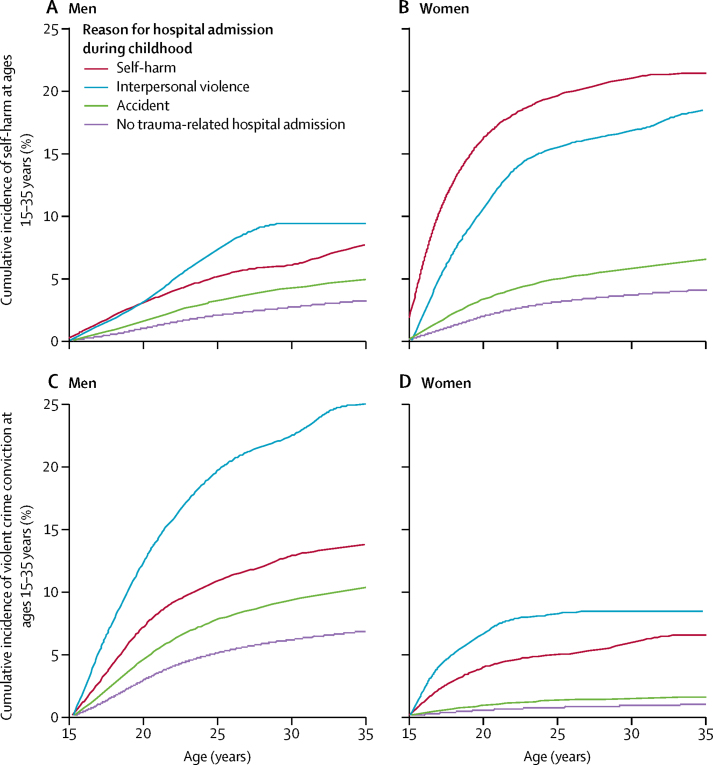
Cumulative incidence (%) for self-harm (men, A; women, B) and violent crime conviction (men, C; women, D) at ages 15–35 years by reason for trauma-related hospital admission before the 15th birthday

The cumulative incidence percentage values shown in table 2 indicate that: (1) for young adult men, the highest absolute risk noted was for violent offending among individuals admitted to hospital for interpersonal violence injury during childhood (cumulative incidence 25·0% [95% CI 21·2–28·9]); (2) for young adult women, absolute risk was highest for subsequent self-harm repetition among those admitted to hospital following self-harm in childhood (cumulative incidence 21·4% [95% CI 19·8–23·1]). Figure 1 shows the substantial sex differences in cumulative incidence (absolute risk) across the 15–35 years age range. Among men admitted to hospital during childhood following interpersonal violence, about a fifth will have committed a violent crime by their 25th birthday and a quarter will have done so by age 35 years (figure 1C). Among women admitted to hospital during childhood after self-harm, almost a fifth will be admitted to hospital again due to self-harm repetition by age 25 years (figure 1B).

**Figure 2:**
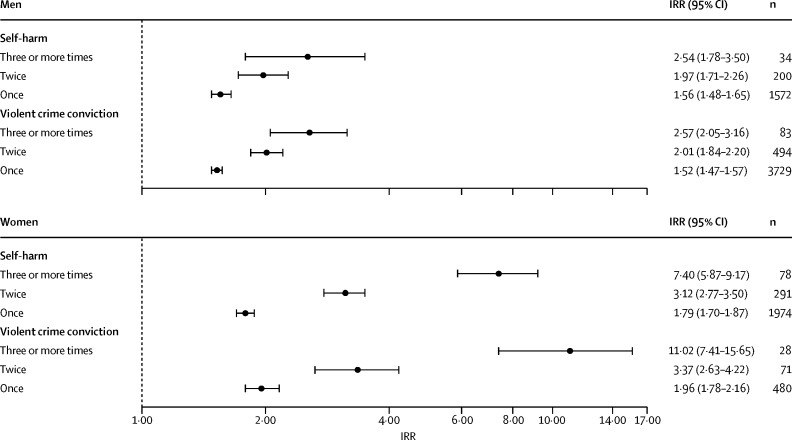
Incidence rate ratios (IRRs)* for self-harm and violent offending at ages 15–35 years according to frequency of trauma-related hospital admissions before the 15th birthday *Reference category for IRR estimates: cohort members who did not experience trauma-related hospital admission before their 15th birthday; IRRs adjusted for age band and calendar year period.

**Figure 3:**
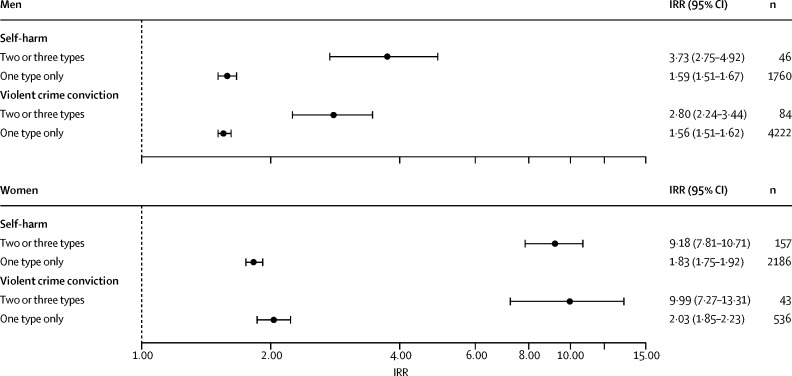
Incidence rate ratios (IRRs)* for self-harm and violent offending at 15–35 years according to number of trauma-related hospitalisation types (self-harm, interpersonal violence, or accident) before the 15th birthday *Reference category for IRR estimates: cohort members who did not experience trauma-related hospital admission before their 15th birthday; IRRs adjusted for age band and calendar year period.

Figure 2 shows the IRRs for later self-harm and violent offending reported separately according to the number of times a cohort member experienced a trauma-related hospital admission during childhood versus the reference category of zero trauma-related hospital admissions. The prevalence of multiple hospital admissions was low in the study cohort (men: admitted twice, n=5528 [1%]; admitted 3 times or more, n=742 [<1%]; women: twice, n=3434 [1%]; 3 times or more, n=427 [<1%]. For both adverse outcomes, we noted an incremental increase in the observed IRR with rising frequency of trauma-related hospital admissions experienced in childhood, and these dose–response relations were much stronger in women than in men: three or more trauma-related hospital admissions (*vs* none) linked with later self-harm risk (women: IRR 7·40 [95% CI 5·87–9·17]; men: 2·54 [1·78–3·50]); three or more trauma-related hospital admissions (*vs* none) linked with later violent criminality risk (women: IRR 11·02 [95% CI 7·41–15·65]; men: 2·57 [2·05–3·16]). Similarly, figure 3 shows IRRs for the two adverse outcomes according to the number of trauma-related hospital admission types during childhood (self-harm, interpersonal violence, or accident). Being admitted to hospital for more than one reason, with comparable exposure prevalence of 0·13% (n=742) in men versus 0·14% (n=765) in women, was associated with substantially greater risks of later self-harm and violent offending compared with one reason only. As with hospital admission frequency, the risk gradients were again far steeper in women than in men: two or three trauma-related hospital admission types (*vs* none) linked with later self-harm risk (women: IRR 9·18 [95% CI 7·81–10·71]; men: 3·73 [2·75–4·92]); two or three trauma-related hospital admission types (*vs* none) linked with later violent criminality risk (women: IRR 9·99 [95% CI 7·27– 13·31]; men: 2·80 [2·24–3·44]).

## Discussion

Incidence rates and cumulative incidence values for self-harm and violent offending were significantly raised among youths and young adults who experienced a trauma-related hospital admission at least once during their childhood. Confounding by parental SES explained very little of these increased risks. In men and women, individuals who were admitted to hospital during childhood following self-harm or interpersonal violence had substantially increased risks for later self-harm and violent criminal offending at ages 15–35 years. Around one in seven men admitted to hospital during childhood following self-harm and one in four due to interpersonal violence will be convicted for committing a violent crime by age 35 years. About one in five women admitted to hospital after an episode of self-harm or interpersonal violence during childhood will be admitted to hospital again following self-harm between their 15th and 35th birthdays. As we expected, the increases in risk linked with hospital admission as a consequence of an accident were far smaller than they were following self-harm or interpersonal violence, probably reflecting a lesser degree of trauma on average and perhaps also lower prevalence of environmental and genetic risk factors among individuals who experienced these episodes. More frequent trauma-related hospital admissions during childhood, and experiencing hospital admissions for multiple types of trauma at such an early age, conferred substantial increases in self-harm and violent criminality risks through late adolescence and young adulthood. Especially steep gradients of this nature were noted among female cohort members.

Our review of the existing published literature indicated that this topic has not been extensively researched, a conclusion that has also been reached by other investigators.^8^ The findings generated from previous studies do, however, largely concur with what we observed. For example, studies done in New Zealand^7^ and Sweden^8^ also showed that history of hospital admission for injury caused by interpersonal violence, as well as previous hospital-treated episodes of self-harm, predict future increased risk of suicidality. Earlier research has reported on subsequent risk of assaultive injury in individuals with a history of hospital admission following self-injury and assault,^6^ but our cohort study is the first to examine links between hospital-treated self-harm occurring before mid-adolescence and later risk of perpetrating violent criminal offence. Consideration of sex-specific absolute risk via cumulative incidence estimates that accounted for competing risks is another distinctive feature of our investigation. Little robust epidemiological evidence exists regarding confounding by familial SES, but the apparent little influence of this phenomenon that we observed concurs with findings reported by a previous national registry study examining links between hospital admission for injury and later suicide risk among Swedish youths.^8^

We believe that the findings generated from this study are likely to be generalisable internationally. Our national cohort study had some major strengths including comprehensive record linkage between multiple registers, absence of recall bias and other sources of information bias, capacity to account for all deaths and emigrations throughout follow-up, nationwide coverage of the registry datasets, and abundant statistical power and precision to examine fairly rare adverse events in a study cohort consisting of more than 1 million people. Thus, for example, our study was entirely free of the linkage errors and resulting selection biases that were reported by a previous study of this topic done using Hospital Episode Statistics in England.^3^ A specific strength of our register-based cohort study was that we could delineate childhood trauma-related hospital admissions to a clinically significant level of severity, because the exposure classification included only those individuals who were actually admitted to hospital. We were also unable to examine emergency department contacts following trauma without admission in this study. However, this should not be regarded as an omission, because such an approach would have identified a much larger set of relatively minor accidental injuries to children, most of whom would be unlikely to have increased risk of experiencing the longer-term adverse outcomes we examined beyond their 15th birthdays.

A widely recognised generic limitation of registry studies is residual confounding.^24^ Thus, adjustment for parental SES took account of only three parameters, and there are many other important determinants of risk, including child abuse and neglect, bullying by peers, and household dysfunction,^25^ that were not measured systematically in the administrative registers that we had access to. Studies of this particular topic share a common limitation that a sizeable proportion of violence and self-harm are undetected at hospital admission and might frequently be misclassified as accidents.^4^ A limitation specific to our cohort study was that we could not examine trauma-related hospital admissions that were of undetermined cause, which was possible in studies done using routinely collected datasets in other countries such as New Zealand^6^, ^7^ and the USA.^4^ This “reason for hospitalisation” category was unavailable to us because of anomalies in registration procedures and absence of consistency in ICD 8th and 10th revision coding usage in the hospital registers across the whole of the study's observation period. Individuals admitted for injuries of undetermined cause have been reported to show substantially increased risk for subsequent suicidal behaviour.^7^ The exclusion of this group might have attenuated some of the relative risk estimates we observed, specifically in table 1 and in Figure 2, Figure 3, albeit only to a marginal degree because this is likely to be an exposure subgroup with rare prevalence. Finally, in this study we did not examine the potential influences of psychopathology among cohort members and also their parents' mental disorders. The latter could have important roles in the cause of trauma-related hospital admission during childhood^26^ as well as self-harm and interpersonal violence during older adolescence and early adulthood.^27^ A comprehensive investigation of these potential causal mechanisms would require a complex study design and a sophisticated analytical approach, ideally with linkage to both primary and secondary care data sources to enable capture of all diagnosed mental illnesses among cohort members and their parents.

Trauma-related hospital admission during childhood could be a useful marker for myriad forms of familial dysfunction and distress that in later years promote emotional dysregulation and impulsive, self-destructive reactions to stress and adversity in adulthood.^28^ In developing multiagency strategies to tackle internalised and externalised violence among susceptible youths and young adults, the wellbeing of individuals who experienced hospital admission during childhood for self-harm or interpersonal violence merits especially close attention. High school and college-oriented programmes might be particularly beneficial in this regard.^29^ National clinical guidelines typically address single problems such as self-harm,^30^ but might need to be broadened to encompass other types of adverse events to encourage family-level psychosocial assessments following hospital admission of children due to serious episodes of interpersonal violence^3^ and accidental injury or poisoning caused by apparent parental neglect. Childhood hospital admissions following trauma might therefore represent crucially important opportunities for hospital-based family-oriented interventions aimed at reducing future risk of accidental, self-inflicted and assaultive injuries, and poisonings.

To conclude, young people who harm themselves or who are aggressive or violent towards others have often experienced trauma during their childhoods, and therefore they should be treated sympathetically rather than as problematic and undeserving. Among those individuals who experienced trauma-related hospital admission during their childhood, risks of adverse outcome are increased, irrespective of parental socioeconomic status. Therefore, a more psychosocial approach to meet the needs of these vulnerable young people and their families is indicated for successful prevention of future episodes of harmful behaviour. The novelty of this study lies in its examination of two associated destructive behaviours for an under-researched exposure-outcome association that spans two adjacent periods in the life-course that are crucially important in an individual's development through to healthy adult maturity. Reporting of sex-specific absolute and relative risks also breaks new ground for this research topic—an approach that maximises the relevance and use of the findings for clinicians, public health professionals, and policy makers
